# Targeted Delivery of Pennyroyal via Methotrexate Functionalized PEGylated Nanostructured Lipid Carriers into Breast Cancer Cells; A Multiple Pathways Apoptosis Activator

**DOI:** 10.34172/apb.2023.077

**Published:** 2023-04-29

**Authors:** Amin Mahoutforoush, Leila Asadollahi, Hamed Hamishehkar, Soheil Abbaspour-Ravasjani, Atefeh Solouk, Masoumeh Haghbin Nazarpak

**Affiliations:** ^1^Immunology Research Center and Students Research Committee, Tabriz University of Medical Sciences, Tabriz, Iran.; ^2^Drug Applied Research Center, Tabriz University of Medical Sciences, Tabriz, Iran.; ^3^Biomedical Engineering Department, Amirkabir University of Technology (Tehran Polytechnic), Tehran 1591634311, Iran.; ^4^New Technologies Research Center (NTRC), Amirkabir University of Technology, Tehran 1591634653, Iran.

**Keywords:** Antioxidants, Breast cancer, Pennyroyal, Methotrexate, PEGylated nanostructured lipid carriers

## Abstract

**Purpose:**

Pennyroyal is a species of the Lamiaceae family with potent anti-cancer and antioxidant properties. Combining this antioxidant with chemotherapeutic agents enhances the effectiveness of these agents by inducing more apoptosis in cancerous cells.

**Methods:**

Here, methotrexate (MTX) combined with pennyroyal oil based on PEGylated nanostructured lipid carriers (NLCs) was assessed. These nanoparticles were physiochemically characterized, and their anti-cancer effects and targeting efficiency were investigated on the folate receptor-positive human breast cancer cell line (MCF-7) and negative human alveolar basal epithelial cells (A549).

**Results:**

Results showed a mean size of 97.4 ± 12.1 nm for non-targeted PEGylated NLCs and 220.4 ± 11.4 nm for targeted PEGylated NLCs, with an almost small size distribution assessed by TEM imaging. Furthermore, in vitro molecular anti-cancer activity investigations showed that pennyroyal-NLCs and pennyroyal-NLCs/MTX activate the apoptosis and autophagy pathway by changing their related mRNA expression levels. Furthermore, in vitro cellular studies showed that these changes in the level of gene expression could lead to a rise in apoptosis rate from 15.6 ± 8.1 to 25.0 ± 3.2 (*P*<0.05) for the MCF-7 cells treated with pennyroyal-NLCs and pennyroyal-NLCs/MTX, respectively. Autophagy and reactive oxygen species (ROS) cellular evaluation indicated that treating the cells with pennyroyal-NLCs and pennyroyal-NLCs/MTX could significantly increase their intensity in these cells.

**Conclusion:**

Our results present a new NLCs-based approach to enhance the delivery of pennyroyal and MTX to cancerous breast tissues.

## Introduction

 The rate of diseases and infections grows with the increasing population. Among the various threats to humans, cancer is responsible for many deaths worldwide, and most of the deaths are due to cancer metastasis.^1,2^ Cancer is defined as an atypical and abnormal condition that leads to a multistage carcinogenic process and targets several cellular physiological systems.^3^ Chemotherapy, radiotherapy, and surgery are the most accepted therapeutic methods for cancer.^4^ The main difficulties of the current cancer therapy approaches are the uncertain distribution of the agents, the insufficient concentration of the drugs, and inadequate monitoring of the drug until it reaches the tumor.^5^ The main reason for the severe side effects of chemotherapeutic agents, such as multiple drug resistance, is deficient drug delivery to target areas.^6^ Therefore, we need to identify therapeutic agents with enhanced targeting capability and high delivery ability. Meanwhile, breast cancer is common cancer in women worldwide.

 Despite the progress ­in various therapeutic methods, the survival rate of patients with breast cancer has not increased significantly.^7^ Conventional chemotherapy agents are spread non-specifically throughout the body and influence both tumor and healthy tissues. This therapeutic approach leads to excessive toxicity in the body.^8^ Therefore, new approaches to breast cancer treatment must be developed to target tumor cells. New achievements in nanotechnology have led to the development of novel targeting plans to increase drug concentrations inside the tumor while restricting systemic side effects and toxicity.^9^ Lipid-based nanoparticles, including liposomes, are highly suitable agent carriers for cancer treatment applications.^10^ Nanostructured lipid carriers (NLCs) are a promising lipid-based drug delivery system with extensive loading capability, prolonged stability, and the ability to release drugs and medications in a controllable fashion at specified rhythmic intervals.^11^ While NLCs are suitable carriers for hydrophobic materials and medicines, they do not load well for hydrophilic drugs such as methotrexate (MTX).^12^ The double emulsion method is a possible way of loading hydrophilic agents in NLCs but causes low drug loading. An excellent approach to this problem, particularly in nanoparticles with a large ratio of surface to mass, is surface ion coordination.^12^ MTX is one of the drugs used to treat different types of cancers, including breast, skin, lung, and head and neck cancers.^13^ MTX inhibits folic acid production, which binds to the dihydrofolate reductase enzyme and consequently hinders dihydrofolate from tuning into tetrahydrofolate. MTX acts not only as a drug but also as a ligand due to its structural similarity to folic acid.^13^ The use of natural ingredients to elevate the synergistic effect of chemotherapy drugs and reduce their side effects is of particular interest. One of the natural ingredients that can inhibit breast cancer cells is pennyroyal oil.^14^

**Figure 1:**
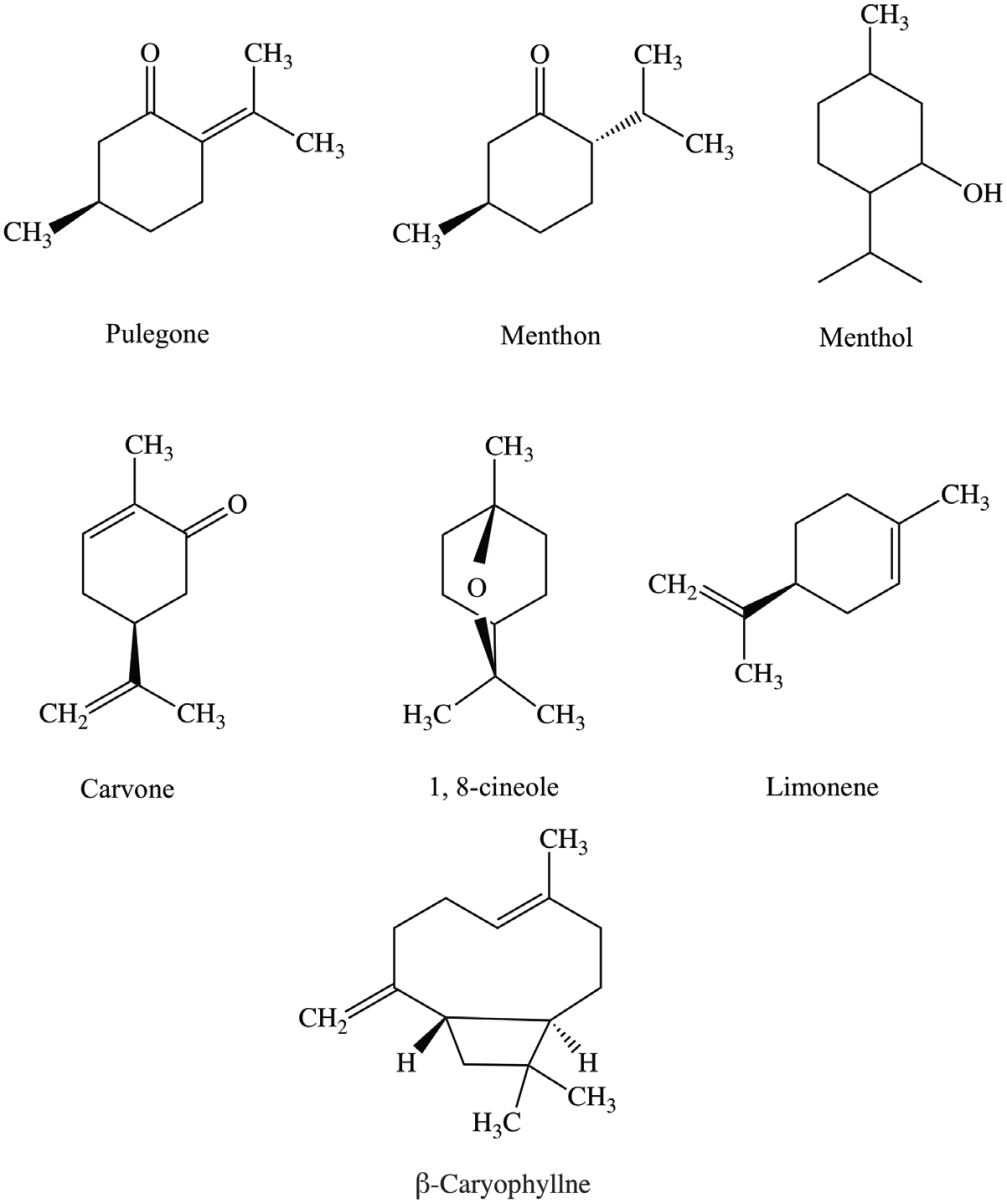
Displays the chemical structure of the seven most important and abundant chemical compounds of pennyroyal oil

 Pennyroyal is a natural herbal extract from Mentha pulegium or Hedeoma pulegioides leaves; mint, family plants are European native.^15^ Pennyroyal oil contains the volatile oil pulegone and other monoterpenes. As is shown in Figure 1, the main active ingredients of the pennyroyal oil are; pulegone, menthone, menthol, carvone, limonene, eucalyptol, and β-caryophyllene. These ingredients give antimicrobial, antioxidant, and anti-cancer effects to the essential oil.^16^ Antioxidants are like double-edged swords; they can reduce oxidative stress at low concentrations, and on the contrary, increasing their engagement can cause oxidative stress.^17^ Due to its antioxidant properties, pennyroyal oil can effectively create free radicals in cancer cells and activate the apoptotic pathway.^18^

 This work aimed to decorate PEGylated NLCs with MTX as nanoparticles for the delivery of pennyroyal in a targeted way and assess the anti-cancer effects of this nano-formulation on the apoptosis, proliferation, cell autophagy, reactive oxygen species (ROS), and colony formation of FR expressing MCF-7 human breast cancer cells.

## Materials and Methods

###  Materials

 Monodansylcadaverine (MDC), 1,2‐Dioleoyl‐3‐trimethylammonium‐propane (DOTAP), Poloxamer 407, 2’,7’-dichlorodihydrofluorescein diacetate (DCFH-DA), DAPI, and 1,2‐dioleoylsn‐glycerol‐3 phosphoethanolamine (DOPE) were purchased from Sigma-Aldrich (St. Louis, Missouri, United States). Caprylic/capric triglycerides (Miglyol^®^ 812) were purchased from Sasol (Witten, Germany), and Precirol^®^ ATO 5 was acquired from Gattefosse (St-Priest, France). DSPE‐mPEG 2000 was obtained from Lipoid GmbH (Ludwigshafen Germany). Moreover, pennyroyal oil was obtained from Barij Essence Pharmaceutical (Mashhad-e Ardahal, Iran), and MTX was provided from Excella GmbH (Feucht, Germany). Also, cell culture materials were obtained from Gibco (Life Technologies Corporation, Thermo Fisher Scientific Inc, Waltham, Massachusetts). Human non-small-cell lung carcinoma cells (A549) and breast cancer cells (MCF-7) were acquired from the Pasteur Institute of Iran (Tehran, Iran).

###  Preparation of pennyroyal-NLCs

 The NLCs were prepared by hot-homogenization (HH) method. First, 20 mg of pennyroyal was dissolved in 100 mg of Miglyol. Then, 100 mg of Precirol was added to the oil mixture and heated up to 80 °C. In the second step, 20 mg of DOTAP: DOPE (1: 1) and 0.1 mg of DSPE-mPEG 2000 were added to the hot lipid mixture and stirred with a homogenizer (5000 rpm) until a uniform lipid solution was obtained. Finally, the surfactant solution (20 mg/mL poloxamer 407 dissolved in phosphate buffer 10 mM pH 5.5) was added slowly and dropwise to the lipid phase under high-speed homogenization (20000 rpm for 20 minutes, Silent crusher M, Heidolph, Nuremberg, Germany). The prepared o/w nanoemulsion was allowed to be cooled down rapidly at room temperature in the refrigerator, leading to the recrystallization of the lipid phase. As a result, the pennyroyal-NLCs were prepared.^19^

###  Preparation of pennyroyal-NLCs/MTX

 DOTAP is a well-known cationic lipid, and DOPE is a helper lipid for DOTAP.^20^ To obtain the best conjugation performance in this study, we used both cationic and helper lipids to prepare NLC. According to the previously published studies, MTX was decorated to the prepared positively charged NLCs by electrostatic conjugation, and the optimal ratio of the decoration was determined.^13^ For electrostatic conjugation of MTX to the surface of the NLCs, 10 mL of pennyroyal-NLCs was added dropwise into 10 mL of MTX solution and mixed without heating at 300 rpm for an hour. Also, to determine the optimal amount of MTX to the NLCs, different ratios of MTX:NLCs (1:3, 1:2, 1:1, 2:1, 3:1, w/w) were used. Then, the best MTX: NLCs ratio was selected based on physicochemical characterization results.

###  Preparation of RhB-NLCs and RhB-NLCs/MTX

 For evaluating the effect of MTX conjugation on the cellular uptake efficacy, red fluorescence-labeled NLCs were prepared by RhB encapsulation into the NLCs. The fluorescence-labeled NLCs were prepared as same as pennyroyal-NLCs.^21^ The only difference in all the preparation stages of the above nanoparticles was the replacement of RhB (at the 0.1 mg/mL concentration) with pennyroyal. Also, unloaded RhB was separated from the nanoparticle solution using the ultrafiltration technique (Amicon^®^ Ultra-4 100 k – a 30 kDa molecular weight cut-off membrane, Merck Millipore).

###  Drug loading (%DL) and encapsulation efficiency (%EE) measurements

 The encapsulation efficiency (EE) of pennyroyal was evaluated using the M_i_/M_o_ × 100 equation, where M_i_ and M_o_ indicate the drug weight before and following the separation, respectively. Moreover, the drug loading ratio was computed as the fraction of the weight of the encapsulated drug to the total weight of the carrier, i.e., lipid. To separate the unloaded pennyroyal oil, the prepared nanoparticles were centrifuged (Universal 320, Pole Ideal Tajhiz Co., Iran) at 5000 rpm for 20 minutes. A sampler collected the surface medium containing the probably unloaded essence. Then, the collected solution was diluted using ethanol. The amount of unloaded pennyroyal was measured using ultraviolet-visible spectroscopy (Ultrospec 2000 Pharmacia Biotech, Cambridge, England) at the wavelength of 281 nm.^11^

###  Size, zeta potential, and morphological characteristics of nanoparticles

 The size, dispersion coefficient, and surface charge of pennyroyal-NLCs and pennyroyal-NLCs/MTX were assessed through dynamic light scattering (DLS; Zeta sizer Nano ZS, Malvern Instruments) at 25 °C.

 Transmission electron microscopy (TEM) was utilized to examine the core-shell structure of nanocarriers. For TEM imaging, 10-fold diluted NLC sample droplet was located on a carbon copper grid and then dried at room temperature. The images were then recorded by TEM (Zeiss NEO-906, 100 KV Germany).

###  Culture and treatment of MCF-7 cell line

 The MCF-7 cells were purchased from the Pasteur Institute of Iran. The cells were defrosted in 7 ml of RPMI-1640 culture medium containing antibiotics and 10% of fetal bovine serum (FBS). By transferring the defrosted cells to a 25 cm^2^ flask and reaching a cell density of over 70%, the cells were detached from the bottom of the flask using 0.25% trypsin, and cell passage was performed on the cells. For cell experiments, third passage cells were used.^13^

###  Sterilization of prepared nanoparticles 

 We used the ethylene oxide purge technique to sterilize the prepared nanoparticles.^22^ The prepared samples were put into a DMB sterilization machine at 1.7 bar pressure for 2.5 hours (37 °C, 85% carbon dioxide plus 15% ethylene oxide, pre-vacuum 20 minutes). After finishing the sterilization process. The liquid samples were sonicated in an ultrasonic bath sonicator for 15 minutes to discharge the remaining sterilization gas.

###  Cell viability assay

 The MTT assay test was utilized to assess the effect of pennyroyal-NLCs and pennyroyal-NLCs/MTX on cell viability. The cells were seeded in a 96-well plate and incubated for 24 hours. Next, the cells were treated with 100 μL of various concentrations (5-1280 μg/mL) of free pennyroyal, pennyroyal-NLCs, and pennyroyal-NLCs/MTX. The plate media was removed after 24 and 48 hours of drug treatment and washed twice with phosphate-buffered saline (PBS). In the next step, a mixture of 150 μL of fresh medium and 50 μL of MTT solution (2 mg/mL in PBS) was added to each well of plates. After four hours of incubating medium with the mixture, 20 μL of Sorensen’s buffer and 180 μL of DMSO were replaced. Eventually, the absorption of the wells was recorded using the Elisa reader (TCAN, Austria) at the wavelength of 570 nm. The reference wavelength was 630 nm.^23^

###  Investigating cell DNA morphology with DAPI staining

 DAPI staining were used to evaluate the degree of DNA damage. For this test, the MCF-7 cells were cultured at 12 mm coverslips at a concentration of 2 × 10^5^ cells per well. After incubation for 48 hours, the cells were treated with IC_50_ concentrations of free pennyroyal, pennyroyal-NLCs, and pennyroyal-NLCs/MTX, obtained from MTT assay results. After 48 hours of treatment, the cells were washed twice with PBS and fixed with 4% formalin for four hours. After the fixation, the cells were rewashed by PBS and treated with Triton X100 solution (0.01% V/V) for 15 minutes. After Triton X100 treatment, the cells were stained with DAPI solution at the concentration of 1 μg/mL. Finally, the stained cells were imaged and evaluated with a fluorescence microscopy system (Olympus, BX50).

###  Evaluation of cell apoptosis and necrosis by annexin V/PI method

 Flow cytometry analysis was utilized to assess the cell death (necrosis or apoptosis) pathway. MCF-7 cells were cultured in a 6-well plate at a concentration of 2 × 10^5^ cells per well. Following 48 hours of incubation, the culture medium was substituted with 2 ml of fresh medium, including free pennyroyal, pennyroyal-NLCs, and pennyroyal-NLCs/MTX at IC_50_ concentrations, and incubated again for 48 hours. Then, the cells were isolated and stained with an annexin V/PI staining kit based on the protocols recommended by the manufacturer. The apoptosis rate was obtained through flow cytometry (BD FACSCalibur^TM^; Becton, Dickinson; NJ) and analyzed using FlowJo software (Tree Star Inc., San Carlos, CA) in each group.^24^

###  Detection of autophagic vacuoles

 MDC is a fluorescent marker used to visualize and identify the autophagy process. MDC was utilized to discover the activation of the autophagy pathway in MCF-7 cells. For this purpose, the cells were cultured at 12 mm coverslips at a concentration of 2 × 10^5^ cells per well. Following incubation for 48 hours, the cultured cells were treated with IC_50_ concentrations of free pennyroyal, pennyroyal-NLCs, and pennyroyal-NLCs/MTX. Following treatment for 48 hours, the cells were washed twice with PBS and stained with 0.05 mM MDC at 37 °C for 10 minutes. The cells were washed three times with PBS to remove excess MDC and assessed using a fluorescence imaging microscope system (Olympus, BX50).^11^

###  Cell uptake 

 For evaluating the effect of active targeting on the cells NLC penetration rate, the RhB-loaded fluorescent formulations were used. The MCF-7 and A549 cells were cultured into 12-well plates and then incubated overnight. Next, the cells were treated with RhB-NLCs and RhB-NLCs/MTX with time intervals of 2, 4, 12, 24, and 48 hours. Then, the penetration rate of the prepared formulations into the cell was measured using the flow cytometry instrument, and the obtained data were analyzed using FlowJo software.^25^

###  Colony formation 

 The colony formation test was conducted to evaluate the ability of the pennyroyal-NLCs, free pennyroyal, and pennyroyal-NLCs/MTX to inhibit colony formation. For this purpose, 2 × 10^3^ cells per well of MCF-7 cells were cultured in a 6-well plate and then incubated for 15 days; after 15 days of incubation, the cell were treated with IC_50_ concentration of the samples and incubated for 48 hours. Finally, the colonies were fixed and stained with ten times diluted solution of crystal violet and then photographed with iPhone 8 (Apple Inc. Cupertino, California, United States) and counted using ImageJ software.^26^

###  Radical scavenging activity measurement

 The ROS technique was used to evaluate the induction rate of intracellular free radicals by analyzing the intensity of 2-7 dichlorodihydrofluorescein- DCFH-DA diacetate fluorescent dye using FACS flow cytometry. MCF-7 cells were cultured in a 6-well plate and then treated with IC_50 _concentrations of free pennyroyal, pennyroyal-NLCs, and pennyroyal-NLCs/MTX obtained from MTT assay results. H_2_O_2_ (100 µM) was used as the positive control. The cells were incubated for 48 hours with these formulations. In the next step, the cells were stained with 500 μL of DCFH-DA (10 μM into PBS) fluorescent dye and incubated for 2 hours. Then, the cells were detached from the plates and washed twice with PBS. Finally, the cells were resuspended into 500 μL of PBS, and their fluorescent intensity was analyzed by flow cytometry using an FL2-H filter.^27^

###  Antioxidant activity and stability measurement

 Antioxidant activity was evaluated in two ways. In the first step, the antioxidant activity of free pennyroyal and pennyroyal-NLCs was examined by measuring the inhibition rate of free radicals using 1,1-diphenyl-2-trinitrobenzene hydrazine (DPPH). In the second, the 2.1 mg/mL concentration was selected to evaluate the antioxidant stability. For this purpose, 5 samples of pennyroyal and pennyroyal-NLCs were prepared at a concentration of 2.1 mg/mL and stored in a dark place during the test. Then, the antioxidant activity of the samples was measured every 15 days up to 60 days by mixing 100 μL of the mentioned samples with 100 μL of DPPH free radical at a concentration of 0.4 mM. The prepared mixtures were poured into a 96-well plate and incubated for 30 minutes in a dark place at room temperature. Finally, the absorbance of the incubated samples was measured at 517 nm (maximum absorption wavelength of DPPH) using a microplate reader.^28^

###  Real-time PCR

 For evaluating the molecular mechanism of prepared nanoparticles against MCF-7 cells, the real-time PCR (RT-PCR) gene expression measurement was done. For RNA isolation from cell plates, the TRIzol^®^ regent was used. In the first step, the MCF-7 cells at the density of 5 × 10^4^ cells per well were seeded into 6 well plate. After 48 hours of incubation, the cells were treated for 48 hours with IC50 concentrations of free pennyroyal, pennyroyal-NLCs, and pennyroyal-NLCs/MTX. For RNA isolation, the treated cells were harvested and mixed with 1 mL of TRIzol solution and incubated for 20 minutes; next 200 μL of chloroform was added to the TRIzol cocktail and mixed gently. The prepared cocktail was incubated in refrigerators (The temperature was settled at -20 °C) for 20 minutes. For separating the TRIzol/chloroform cocktail layers, the cocktail was centrifuged at 12 000 RPM speed at 4–8 °C for 15 minutes. Finally, colorless upper-phase media was harvested, gently mixed with 500 μL of isopropyl alcohol, and incubated for 10 minutes. For depositing the extracted RNA, the prepared cocktail was centrifuged at 12 000 RPM for 10 minutes. Finally, the formed precipitate, for better drying efficacy, was washed three times with isopropyl alcohol and dried at room temperature. The isolated RNA was dissolved in 20 μL RNase free water, and the RNA concentration was measured by a Nano-Drop spectrophotometer (2000, Thermo Fisher Scientific Life Sciences, Waltham, Massachusetts). The complementary cDNA was generated by the QuantiTect Reverse Transcription cDNA synthesis kit (Qiagen, Hilden, Germany). RT-PCR was done via the Power SYBR GREEN Master Mix (Applied Biosystems, Foster City, California). β-Actin was amplified as a reference gene. mRNA expression was measured with the 2− ΔΔCT method. Table 1 addressed the primer sequences used in RT-PCR reactions.^13^

**Table 1:** Primer sequences

**Gene **	**Forward**	**Reverse**
β-Actin	5′-AGCACAGAGCCTCGCCTT-3′	5′-CATCATCCATGGTGAGCTGG-3′
Bcl-2	5′-CCTGTGGATGACTGAGTACC-3′	5′-GAGACAGCCAGGAGAAATCA-3′
ERK 1	TGGCAAGCACTACCTGGATCAG	GCAGAGACTGTAGGTAGTTTCGG
ERK 2	ACACCAACCTCTCGTACATCGG	TGGCAGTAGGTCTGGTGCTCAA
Survivin	CCACTGAGAACGAGCCAGACTT	GTATTACAGGCGTAAGCCACCG
Caspase 3	GGAAGCGAATCAATGGACTCTGG	GCATCGACATCTGTACCAGACC
Caspase 8	AGAAGAGGGTCATCCTGGGAGA	TCAGGACTTCCTTCAAGGCTGC
Caspase 9	GTTTGAGGACCTTCGACCAGCT	CAACGTACCAGGAGCCACTCTT
Bax	TCAGGATGCGTCCACCAAGAAG	TGTGTCCACGGCGGCAATCATC
P-53	5′-CCTCAGCATCTTATCCGAGTGG-3′	5′-TGGATGGTGGTACAGTCAGAGC-3′
P-38	5′–AGAGTCTCTGTCGACCTGCT-3′	5′-CCTGCTTTCAAAGGACTGGT-3′

###  Statistical analysis

 All the experiments were repeated three times in a row. The two-way analysis of variance (two-way ANOVA) and independent *t* test were employed in the statistical analysis, including multiple comparisons between confession data using the Tukey honest noteworthy difference test (GraphPad Prism, version 8, San Diego, CA). A significant *P* value of less than 0.05 was used, and the FlowJo software package was used to analyze the data obtained by flow cytometry (V10.5.3, Tree Star Inc., San Carlos, CA).

## Results and Discussion

###  Preparation and characterization of NLCs

####  Preparation and PEGylation of NLCs

 The lipid phase was composed of Precirol, pennyroyal, DOTAP, DSPE-mPEG 2000, and DOPE. The presence of PEG on the surface of lipid nanovesicles extends the circulation lifetime of the vehicle in the body’s bloodstream.^29^ The role of DOPE and DOTAP is to provide cationic charge for lipid nanoparticles,^30^ leading to the formation of MTX-loaded NLCs through electrostatic conjugation between MTX with a negative charge and nanoparticles with a positive charge.^31^ Therefore, only MTX was utilized for conjugation onto the surface of the nanoparticles because of cationic lipids, DOPE, and DOTAP. Also, it should be noted to fully benefit from targeting the performance of MTX; we tried to minimize the positive charge of the NLCs to reduce the positive effect of charge in nanoparticle penetration to the surface of negatively charged normal cells.

####  Surface decoration of PEGylated nanoparticles with MTX

 The Precirol:DOPE:DOTAP:DSPE-mPEG 2000 was prepared a 10:1:1:0.1 weight ratio, and NLCs:MTX was prepared a 10:1 weight ratio. The optimal proportion of the phospholipids to the drug and the nanoparticles was determined based on our pilot study results. The nanoparticles were produced and described by determining size, the polydispersity index, zeta potential, EE, and drug loading. The *in vivo* destiny of the prepared nanoparticles might be influenced by multiple physicochemical factors such as surface charge and size. Based on studies examining the effects of nanoparticle size on biological and pharmacokinetic properties, the optimal size of nanoparticles is in the range of 130 to 300 nm for drug delivery purposes.^11^ Nanoparticles within this size range are more durable in the circulatory system than free drugs since they can efficiently bypass the reticuloendothelial system. It should be noted that nanoparticles larger than 300 nm are eliminated by the reticuloendothelial system.^11^

####  Physicochemical characterization of nanoparticles

 The DLS method was utilized to exhibit zeta and size alterations in different stages of the preparation of nanoparticles. It was also used to confirm the kind of conjugations. Based on the observations, pennyroyal was encapsulated into NLCs successfully, and the zeta potential and average size of the prepared NLCs were + 39.0 ± 6.9 mv and 97.4 ± 12.1 nm, respectively (Table 2). Also, according to our DLS results, the PDI values of the non-targeted and targeted NLCs were 0.444 and 0.836, respectively. Furthermore, the percentage values of EE and DL for pennyroyal were 96.0 ± 6.0% and 9.6 ± 0.6%, respectively. In addition, the conjugation of MTX to the NLCs was conducted using an electrostatic conjugation technique based on the surface charge of the ingredient. For the electrostatic conjugation, zeta potential and size were determined among MTX and NLCs. The DLS technique was utilized for evaluating and comparing the zeta potential and size of both NLCs and NLCs-MTX. The particle size and zeta potential of the prepared formulations are shown in Table 2. As shown, the particle size increased with the addition of MTX. Based on the results, binding MTX to the nanoparticles by electrostatic bonding changed the surface charge of NLCs from + 39 ± 6.8 mV to + 1.36 4.4 mV and the size of the nanoparticles from 97.4 ± 12.1 nm to 220.4 ± 11.4 nm (Table 2).

**Table 2:** The composition, particle size, zeta potential, polydispersity index (PDI), encapsulation efficiency (EE), and drug loading (DL) of pennyroyal-NLCs and pennyroyal-NLCs/MTX

**Samples**	**Size (nm)**	**Zeta (mV)**	**PDI**	**%EE**	**%DL**
Pennyroyal-NLCs	97.4 ± 12.1	39.0 ± 6.9	0.444	96.0 ± 6.00^*^ 97.5 ± 3.3^#^	9.6 ± 0.60^*^ 0.97 ± 0.03^#^
Pennyroyal-NLCs/MTX	220.4 ± 11.4	1.36 ± 4.4	0.836		

Pennyroyal:* Mentha pulegium* L. essential oil; NLCs: nanostructured lipid carriers; MTX: methotrexate. The results were calculated as the mean ± standard deviation (n = 3). ^*^ and ^#^ represents %DL and %EE of pennyroyal and MTX respectively.

**Figure 2:**
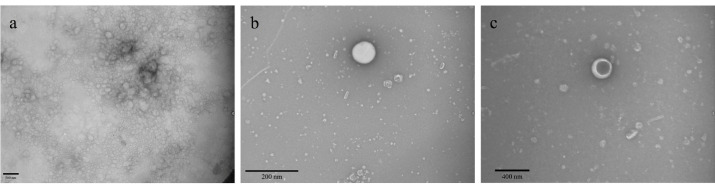
TEM visualizing the morphology of pennyroyal-NLCs (a and b), pennyroyal-NLCs/MTX (c). The data indicate mean ± SD (n = 3). NLCs: nanostructured lipid carriers, MTX: methotrexate, TEM: transmission electron microscopes, SD: standard deviation

 We used TEM to examine the shell and core of the nanoparticles (Figure 2). As shown in Figure 2a, a panorama view of the pennyroyal-NLCs is displayed; this figure confirms the spherical shape of the NLCs and the absence of aggregation in the sample. Also, by comparing Figure 2b with Figure 2c, the MTX surface covering on pennyroyal-NLCs confirmed. MTX was darker nearby the core, the shell was covered with MTX, and the central core was dark.

###  Nanoformulation influence on cell cytotoxic activity

**Figure 3:**
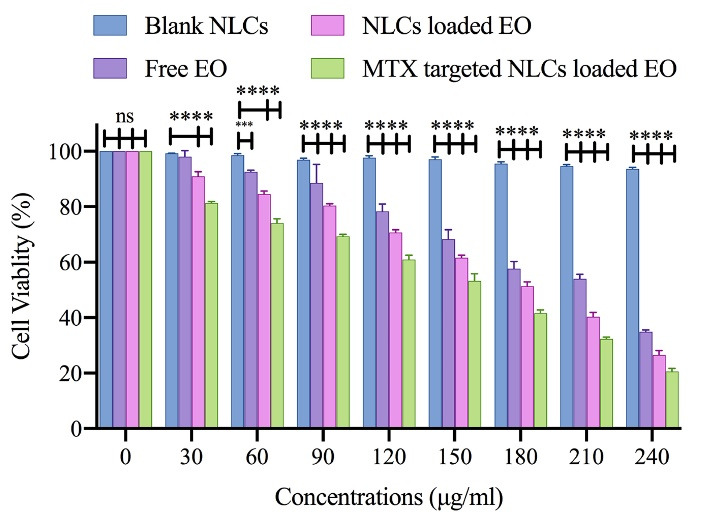
The cell toxicity of free pennyroyal, Blank NLCs, pennyroyal-NLCs, and pennyroyal-NLCs/MTX against MCF-7 breast cancer cells over 48 h. The data indicate mean ± SD (n = 3); ***P < 0.001، and ****P < 0.0001, versus multiple comparisons. NLCs: nanostructured lipid carriers, MTX: methotrexate, SD: standard deviation

 We used the MTT test to assess the cytotoxic effects of free MTX, free pennyroyal, NLCs, and NLCs-MTX on the survivability of the MCF-7 cells. The results showed that cell survivability was significantly reduced following 48 hours treatment of the MCF-7 cells. After 48 hours incubation of the MCF-7 cells, the IC_50_ values for free pennyroyal and pennyroyal-NLCs were 286.1 and 200.8 µg/mL, respectively (Figure 3). The reason can be the superiority of NLCs that elevated drug bioavailability.^19^ Pennyroyal-NLCs targeting via MTX significantly increased, and thus the IC_50 _concentration decreased from 200.8 to 138.7 µg/mL. The MTT test showed that both nanoparticles (targeted and non-targeted) resulted in a considerable reduction in cell survivability compared to the control cells. There was a substantial difference between the cytotoxicity of pennyroyal-NLCs and pennyroyal-NLCs/MTX. The pennyroyal-NLCs/MTX showed better cytotoxic features on MCF-7 cells compared to free pennyroyal and pennyroyal-NLCs. However, pennyroyal-NLCs and pennyroyal-NLCs/MTX affected the survivability of MCF-7 cells differently. These results are consistent with the results obtained in the cellular uptake experiment, revealing the increased cellular uptake of MTX-loaded NLCs relative to NLCs on MCF-7 cells. Recent studies have demonstrated superior binding affinity to folate receptors because MTX decorates NLCs. As a result, MTX-loaded NLCs can recognize folate receptors and internalize them in cells through endocytosis with the mediation of receptors.^13^ Furthermore, NLCs can penetrate cells through nonspecific endocytosis.^12^ Therefore, it can be concluded that NLCs actively targeted by MTX attachment to folate receptors result in better cytotoxic function and better selectivity toward MCF-7 cells, which is consistent with a recent study.^13^

###  Cellular uptake of NLCs

**Figure 4:**
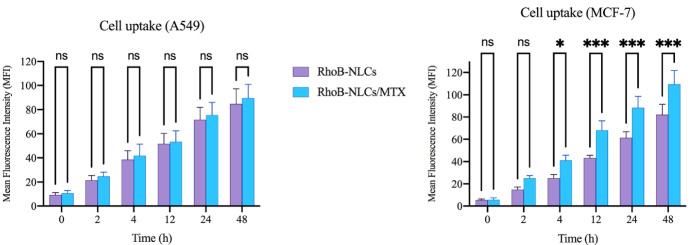
The cellular uptake of high (MCF-7) and low (A549) folate receptor expressed cells treated by RhB-NLCs and RhB-NLCs/MTX. The data indicate mean ± SD (n = 3); ****P < 0.0001, versus multiple comparisons. RhoB: rhodamine B, NLCs: nanostructured lipid carriers, MTX: methotrexate, SD: standard deviation, and NS: not significant

 The cellular uptake of RhoB-NLCs and RhoB-NLCs/MTX has been assessed quantitatively through flow cytometric analysis on A549 cells (slight folate receptor expression) and MCF-7 cells (overexpressing folate receptors). The corresponding fluorescent intensity of RhoB was evaluated to quantify the number of NLCs exposed to internalization. The findings revealed that the two nanoparticles were internalized into MCF-7 cells successfully in a time-dependent fashion, and the uptake of NLCs/RhoB-MTX was significantly larger than that of RhoB-NLCs (*P* < 0.001) (Figure 4). Cellular uptake was also observed following the incubation of A549 by RhoB-NLCs/MTX and RhoB-NLCs. However, there was no significant difference in the cellular uptake of A549 between non-targeted (RhoB-NLCs) and targeted (RhoB-NLCs/MTX) nanoparticles (Figure 4). The findings illustrated the successful decoration of MTX on the surface of NLCs, showing that the cellular uptake of MTX-loaded NLCs relied on folate receptors and facilitated the endocytosis mechanism (i.e., active targeting).^11^ Folate receptors overexpression in the surface of the MCF-7 cells leads to NLCs receptor-based binding to the surface of cells and eventually leads to an increased connection of RhoB-NLCs/MTX to folate receptors and elevated cell uptake.^11^ Receptor-mediated folate endocytosis was the foundation for the cellular uptake of RhoB-NLCs/MTX with MCF-7 cells. However, the primary contributing mechanism might be the nonspecific internalization related to the cell uptake of RhoB-NLCs/MTX and RhoB-NLCs by A549 cells. A recently published article by Abedi et al showed that targeting HMGA-2 siRNA-loaded poly(amidoamine) dendrimers with MTX could significantly increase the cell entrance of HMGA-2 siRNA into MCF-7 cells. Also, their results showed that the surface modification of dendrimers with MTX could actively target the folate receptors of MCF-7 cells. This conclusion was reached when the cell uptake study on A549 cells showed no difference in the fluorescence intensity of non-targeted and MTX-targeted dendrimers.^13^

###  Targeted NLCs increasing apoptosis in cancerous breast cells

**Figure 5:**
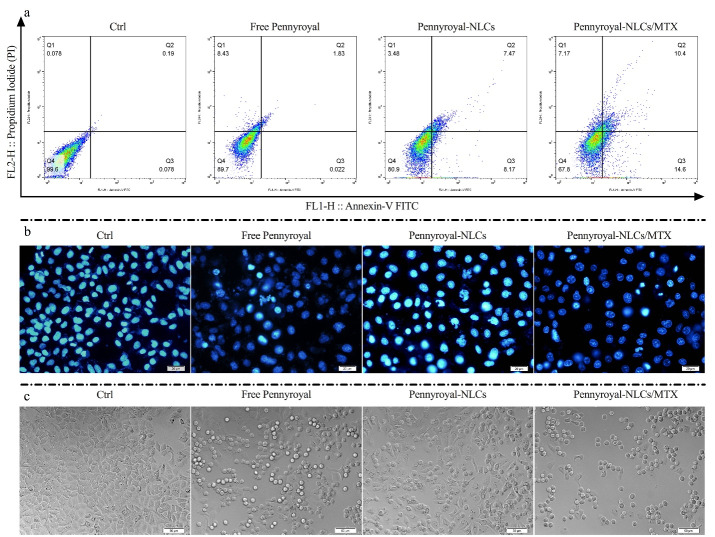
Cell apoptosis induced by free pennyroyal, pennyroyal-NLCs, and pennyroyal-NLCs/MTX in MCF-7 breast cancer cells. Annexin V/PI staining in free pennyroyal, pennyroyal-NLCs, and pennyroyal-NLCs/MTX over 48 h showed a high apoptosis rate in the pennyroyal-NLCs/MTX group (a). Fluorescent images of the treated MCF-7 cells stained with DAPI (b). DIC images of the treated MCF-7 cells (c). NLCs: nanostructured lipid carriers, MTX: methotrexate, DAPI: 2‐(4‐amidinophenyl)‐6‐indolecarbamidine 4′,6-Diamidine-2′-phenylindole dihydrochloride, DIC: Differential Interference Contrast. The images were taken at 40X magnification

 Apoptosis in the MCF-7 cells was quantitatively examined by staining them with annexin V/PI after incubation with free pennyroyal, pennyroyal-NLCs, and pennyroyal-NLCs/MTX for 48 hours. Here, a negative control group was considered to receive blank NLCs treatment. As shown in Figure 5a, apoptosis rates of free pennyroyal and pennyroyal-NLCs were 2 and 15.6%, respectively. Our results showed that the encapsulation of pennyroyal with NLCs decreased the necrosis rate from 8.4% to 3.4%. In a study by Abedi et al, the effect of the loading erlotinib (ELT) on NLCs and liposomes was compared. They reported that loading ELT on the boot of nanocarriers could significantly reduce the A549 necrosis rate compared to free ELT.

 On the other hand, NLCs-loaded ELT has better anti-cancer activity than liposomes-loaded ELT.^12^ Eventually, pennyroyal-NLCs targeting via MTX increased the 60.25% apoptosis rate as compared to non-targeted pennyroyal-NLCs (Figure 5a). Thus, NLCs/MTX nanoparticles induced a higher apoptosis rate than non-targeted nanoparticles (*P* < 0.05) and free forums of the drugs (*P* < 0.001). Apoptosis rates were also assessed qualitatively via the DAPI staining test to identify nuclear fragmentation within the processed MCF-7 cells (Figure 5b). Also, according to our obtained results from DIC imaging, the number of granule cells increased by increasing the apoptosis rate and DNA deformation (Figure 5c). This assay revealed that free pennyroyal and pennyroyal nanoformulation caused fewer nuclear fragmentations and deformations. Nuclear fragmentations increased following the treatment of cells through targeted nanoformulations. Using the receptor base targeting strategy in the drug delivery system is a popular method. In a study by Mansoori et al, hyaluronic acid (HA) was used for targeting 5–fluorouracil (5-FU) loaded cationic liposomes. Their results revealed that targeting the liposomes through HA could significantly raise the apoptosis and deformed DNA numbers as compared to the non-targeted type of 5-FU loaded liposomes.^31^

###  Effect of pennyroyal-NLCs targeting with MTX on MCF-7 cells colony formation

**Figure 6:**
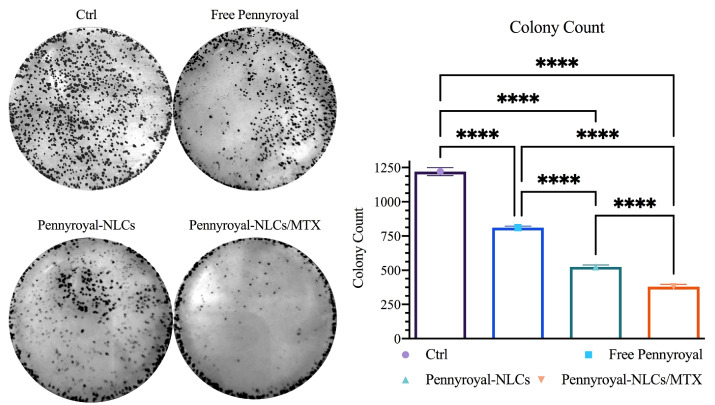
Pennyroyal loaded NLCs blocking clonogenicity in MCF-7 cancerous breast cells. pennyroyal-NLCs, free pennyroyal, and pennyroyal-NLCs/MTX reduced the formation of breast cancerous cell colonies. The data indicate mean ± SD (n = 3); ****P < 0.0001, versus multiple comparisons. NLCs: nanostructured lipid carriers, MTX: methotrexate, and SD: standard deviation

 A clonogenic assay was conducted on MCF-7 cells to determine whether free pennyroyal, pennyroyal-NLCs, and pennyroyal-NLCs/MTX could reduce cell migration and colony formation. The results demonstrated a significant reduction in the colony numbers of cells subjecting to treatments by pennyroyal targeted and non-targeted nanoformulations compared to free pennyroyal and blank NLCs (Figure 6). By looking closely at the number of colony populations, it can be seen that the encapsulation of pennyroyal in NLCs has reduced the colony counts up to 64%. On the other hand, targeting NLCs with MTX has reduced the colony counts by up to 72% compared to non-targeted lipid pennyroyal-NLCs. Accordingly, a recent study exhibited reduced colony formation in the MCF-7 cell line by targeting folate receptors through surface-modified liposomal nanoparticles with MTX, encapsulating gemcitabine.^32^ Mahoutforoush et al in their study showed that targeting docetaxel and doxorubicin-loaded NLCs with MTX significantly reduced the colony count compared to the non-targeted type of doxorubicin and docetaxel-loaded NLCs.^11^

###  Co-delivery of pennyroyal and MTX increasing ROS level

**Figure 7:**
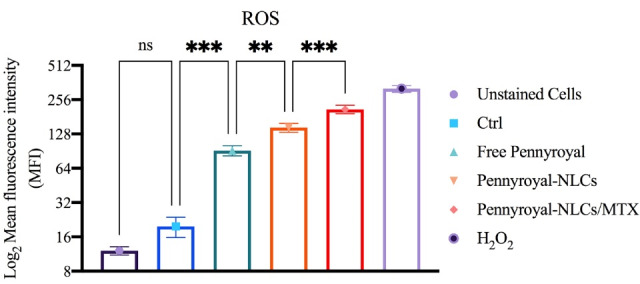
The flow cytometry analysis of ROS induction in MCF-7 cells following treatment by blank NLCs (Ctrl), free pennyroyal, pennyroyal-NLCs, and pennyroyal-NLCs/MTX. The data indicate mean ± SD (n = 3); **P < 0.01 and ***P < 0.001, versus multiple comparisons. ROS: reactive oxygen species, NLCs: nanostructured lipid carriers, MTX: methotrexate, SD: standard deviation, and NS: not significant

 Three pathways are involved in cancer cell death: necrosis, autophagy, and apoptosis.^1^ Immoderate ROS may cause apoptosis via intrinsic and extrinsic pathways. MTX and pennyroyal can result in apoptosis in cancer cells through ROS activation. Here, ROS was assessed through flow cytometry analysis to investigate the active targeting effect of pennyroyal by MTX (Figure 7). The results revealed that the delivery of pennyroyal significantly increased the ROS level as opposed to the free form of pennyroyal. Furthermore, the targeted co-delivery of pennyroyal almost doubled the ROS fluorescence intensity level. Generally, the co-delivery of pennyroyal can increase the ROS fluorescence intensity level, and targeted co-delivery is an efficient way to enhance ROS production. These observations can be ascribed to the significant accumulation of intracellular drugs because of active targeting.^11,17^

###  Molecular cytotoxicity studies

**Figure 8:**
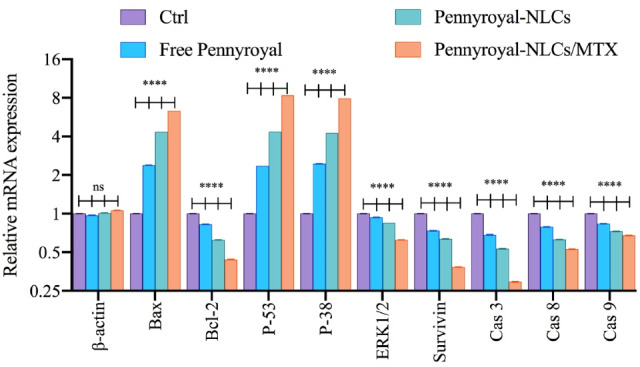
mRNA levels of β-actin, Bax, Bcl-2, P-53, P-38, ERK ½, Survivin, caspase-3, caspase-8, and caspase-9 on MCF-7 (breast cancer) cells treated by blank NLCs (Ctrl), free pennyroyal, pennyroyal-NLCs, and pennyroyal-NLCs/MTX. The data indicate mean ± SD (n = 3); ****P < 0.0001, versus multiple comparisons. NLCs: nanostructured lipid carriers, MTX: methotrexate, SD: standard deviation, and NS: not significant

 As well as we evaluated the gene expression level of apoptosis, autophagy, ROS, and cell migration-related genes by real-time PCR. As shown in Figure 8 cells treated with blank NLCs displayed no noteworthy changes in targeted gene expressions. Moreover, results showed that treating the cells with Free pennyroyal, pennyroyal-NLCs, and pennyroyal-NLCs/MTX leads to decreasing the gene expression levels of Bcl-2, Erk 1/2, survivin, caspase 3, caspase 8, and caspase 9. Statically comparisons showed that targeting the pennyroyal with MTX decreases the expression of the genes as mentioned above significantly as compared to free pennyroyal, and pennyroyal-NLCs. Next, we evaluated the expression level of Bax, P-53, and P38 genes. Our results showed that pennyroyal, could notably increase these gene expression levels in MCF-7 cells; also, the statistical comparisons once again showed that; targeting the pennyroyal with MTX could significantly affect the gene expression level as compared to Free pennyroyal, and pennyroyal-NLCs. The most critical issue in the introduction of a new anticancer agent is the reorganization of the apoptosis pathways by alteration of the levels of apoptotic and anti-apoptotic genes. in the present study, the mRNA expression level of the caspase-8, as an apoptosis promoter, significantly decreased, and simultaneously, the mRNA expression level of apoptotic and autophagic inducers, such as Bax, p-53, and p-38 increased. Increasing the levels of caspase-3 and caspase-8 confirmed the apoptosis messengers’ waterfall activation.^10,17^

 Following, significantly decreasing the mRNA expression level of Survivin, Bcl-2, and Erk 1/2 as anti-apoptotic regulator genes proved the anticancer action of pennyroyal and the superiority of pennyroyal-NLCs/MTX by reducing anti-apoptotic gens expressions and increasing the levels of apoptotic genes.^17^

###  Targeting the pennyroyal-NLCs with MTX increase the autophagy intensity in MCF-7 cells

**Figure 9:**
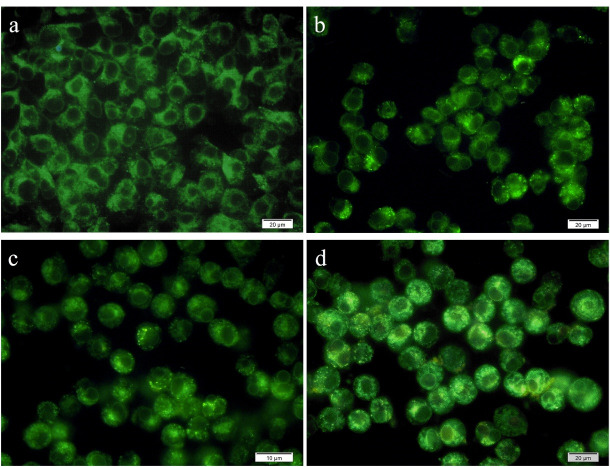
Autophagy at MCF-7 cells following treatment with blank NLCs (a), free pennyroyal (b), pennyroyal-NLCs (c), and pennyroyal-NLCs/MTX (d). NLCs: nanostructured lipid carriers, and MTX: methotrexate. The fluorescent images were taken at 40X magnification

 Autophagy was qualitatively explored by staining MCF-7 cells using MDC (an autofluorescent compound that labels autophagic vacuoles specifically) after 48 hours incubation with free pennyroyal, pennyroyal-NLCs, and pennyroyal-NLCs/MTX. In addition, a negative control group was considered to receive blank NLCs treatment. As shown in Figure 9, MCF-7 cells treated with pennyroyal (Figure 9b), pennyroyal-NLCs (Figure 9c), and pennyroyal-NLCs/MTX (Figure 9d) for 48 hours showed a punctate pattern in MDC-labeled fluorescence, as compared to the control cells that showed a diffuse MDC staining. The results showed that pennyroyal-NLCs/MTX could cause autophagy compared to the single types of them. Moreover, targeting pennyroyal increased autophagic vacuoles in contrast to non-targeted combined nano complex. Also, targeting nanoparticles raised cellular uptake and the chemotherapeutic effect on cancer cells.^31^ Chemoresistance is the main problem in the treatment process of cancer, and autophagy may play an essential role in the diminution of chemoresistance.^33^ There is some evidence to support the role of autophagy in enhancing the effects of radiotherapy and chemotherapy.^33^

###  Evaluation of antioxidant activity

**Figure 10:**
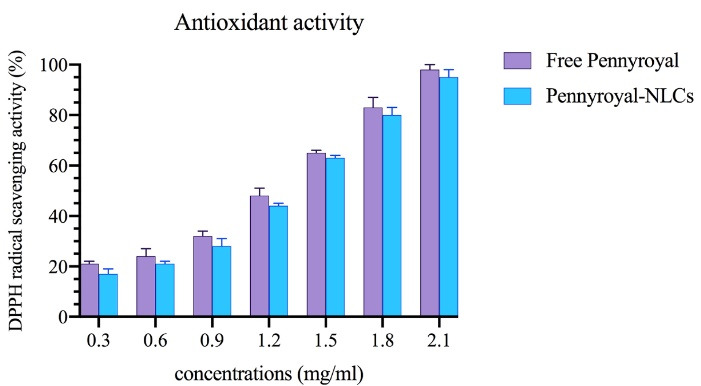
The antioxidant activity of free pennyroyal and pennyroyal-NLCs in different concentrations (0.3 to 2.1 mg/mL) measured by DPPH. The data represent mean ± SD (n = 3). NLCs: nanostructured lipid carriers, and DPPH: 2,2-diphenyl-1-picrylhydrazyl

 The DPPH assay was done to assess the effect of encapsulation on the antioxidant activity of pennyroyal. Figure 10 shows the percentage of inhibition of free pennyroyal and pennyroyal-NLCs by free radicals. According to Figure 10, the antioxidant activity of free pennyroyal was higher than that of pennyroyal-NLCs. In addition, the antioxidant activity showed a dose-dependent flow. The insignificantly lower antioxidant activity of pennyroyal-NLCs compared to free pennyroyal can be explained by the thermal degradation of pennyroyal’s sensitive antioxidant compounds during the preparation process of NLCs. Finally, the IC_50_ values for free pennyroyal and pennyroyal-NLCs were 1.458 mg/mL and 1.136 mg/mL, respectively. In a study by Radbeh et al, the effect of encapsulation on the antioxidant activity of Cornus mas extract was investigated.^34^ Their findings demonstrated that the encapsulation of the herbal extract could decrease its antioxidant activity. They attributed this phenomenon to heating the extract during the preparation of nanoparticles.^34^ Also, our previously published research showed that heating could decompose a few amounts of heat-sensitive compounds.^11^

###  Antioxidant stability study

**Figure 11:**
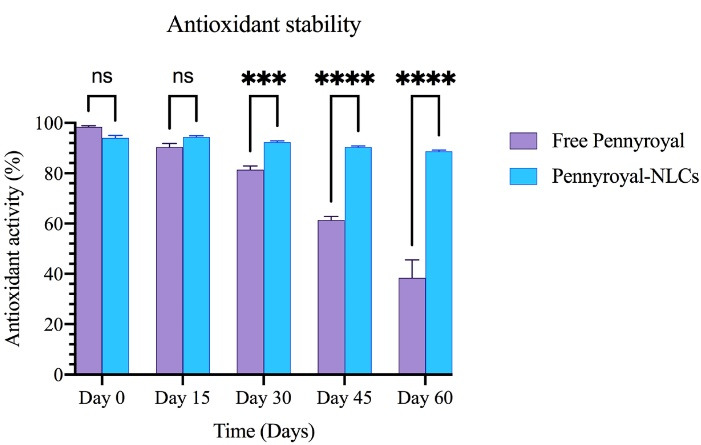
The antioxidant stability of free pennyroyal and pennyroyal-NLCs during storage (0, 15, 30, 45, and 60 days) measured by DPPH. The data indicate mean ± SD (n = 3); ***P < 0.001 and ****P < 0.0001, versus multiple comparisons. NLCs: nanostructured lipid carriers, DPPH: 2,2-diphenyl-1-picrylhydrazyl, and NS: not significant

 Following the oral administration, the bioavailability of antioxidant compounds was controlled by crossing the epithelial tissue of the gastrointestinal tract pathway and appearing in systemic circulation. Antioxidants are degraded due to multiple factors during the transition.^35^ Hence, they may not exhibit their biological effects, such as antioxidant activity, appropriately. pennyroyal encapsulation through NLCs can preserve antioxidants from biodegradation. The DPPH test was conducted to assess antioxidant activity in pennyroyal-NLCs and free pennyroyal throughout 60 days of storage. The results showed a free radical scavenging capacity in a time-dependent manner, but no considerable scavenging activity of NLCs was found (Figure 11). Based on our findings, on the first day, the antioxidant activity of pennyroyal-NLCs and free pennyroyal was 94% and 98.3%, respectively. However, these amounts changed throughout the storage period (i.e., 60 days). Free pennyroyal lost 60% of its antioxidant activity, whereas pennyroyal-NLCs lost 6% of its activity. Stability measurements of antioxidant compounds showed that encapsulation could maintain 94% of the antioxidant aggregation of free pennyroyal. This finding is in line with previous results reported in the studies of Homoki et al^36^ and Kähkönen et al.^37^ As shown in Figure 11, pennyroyal-NLCs showed a considerable antioxidant protective effect. These findings highlight the importance of the carrier by benefiting from the stability resulting from the encapsulation of pennyroyal into a lipid-based nanostructured system. Our results are consistent with those of previous studies.^38,39^

## Conclusion

 In this study, MTX-targeted PEGylated NLCs were formulated with the hot homogenization method using five lipids containing Precirol, DSPE-mPEG 2000, DOPE, DOTAP, and Miglyol-812 for pennyroyal targeted delivery. The results showed that pennyroyal was efficiently encapsulated into PEGylated NLCs, and MTX was well-placed onto the surface of NLCs. In vitro studies have demonstrated that MTX (a targeting ligand) can elevate pennyroyal cellular permeability. As a result, it can reduce the IC_50_ concentration and, in turn, activate the apoptosis, autophagy, and ROS molecular pathways and increases their intensity. Moreover, the clonogenicity evaluation showed that the efficient, targeted transmission of pennyroyal could prevent colony formation in MCF-7 cells. Our antioxidant stability results indicated that the encapsulation of pennyroyal could effectively protect it against harmful environmental conditions. Our findings demonstrate that targeted delivery of nano-pennyroyal and MTX can be considered a promising approach for treating breast cancer.

## Competing Interests

 All the authors of this article declare that they have no conflict of interest.

## Ethical Approval

 In the dissemination of our research findings, we uphold the highest standards of publication ethics. This encompasses the transparent disclosure of any potential conflicts of interest and the rightful attribution of prior work and contributions from collaborators. Our paramount commitment revolves around the safety and well-being of researchers, study participants, and the environment. To uphold this commitment, all members of our laboratory team receive comprehensive training in secure laboratory procedures. We rigorously implement safety precautions when handling cell cultures, hazardous substances, or equipment.
